# Draft Genome Sequence of Scytalidium lignicola DSM 105466, a Ubiquitous Saprotrophic Fungus

**DOI:** 10.1128/MRA.01208-18

**Published:** 2018-10-11

**Authors:** Enrico Büttner, Anna Maria Gebauer, Martin Hofrichter, Christiane Liers, Harald Kellner

**Affiliations:** a Department of Bio- and Environmental Sciences, International Institute Zittau, Technische Universität Dresden, Zittau, Germany; Broad Institute of MIT and Harvard

## Abstract

Scytalidium lignicola is a ubiquitous anamorphic ascomycete and belongs to a genus that includes several phytopathogenic fungi. The strain sequenced in this study (DSM 105466) was isolated from leaves of Quercus robur. The draft genome has a size of 47.7 Mb and contains 12,795 protein-coding genes.

## Abstract (precis)

Scytalidium lignicola is a ubiquitous anamorphic ascomycete and belongs to a genus that includes several phytopathogenic fungi. The strain sequenced in this study (DSM 105466) was isolated from leaves of Quercus robur.

## ANNOUNCEMENT

The dark-walled “melanized” mold Scytalidium lignicola belongs to a genus of widespread anamorphic ascomycetes. Species within that genus generally lack a sexual state (*fungi imperfecti*). They are difficult to assign within the fungal systematics of Eumycota (*incertae sedis* within the order Helotiales), and they are known to cause certain plant diseases, e.g., in Citrus and Manihot (1–3). Recently, several species previously classified within the genus Scytalidium have been classified under a new genus, Neoscytalidium. Representatives of both genera are morphologically rather similar, with the exception of Neoscytalidium hyalinum (= N. dimidiatum), which is a botryosphaeriaceous fungus forming Scytalidium- and Fusicoccum-like synanamorphs under special conditions (4, 5). While colonizing wood, soil, or compost environments, S. lignicola is recognized as a saprotrophic opportunist whose lifestyle can change to human pathogenicity (6, 7). Indeed, little information is available on the role of this facultative pathogen in lignocellulose decomposition, on its interactions with the environment, and on the secretion of cellulose- and lignin-modifying enzymes. By analyzing the genome of S. lignicola, we have gained first insights into these topics.

S. lignicola DSM 105466 (ribosomal cistron GenBank accession number MG815782) was isolated from leaf litter pieces of Quercus robur (Cottbus, Germany, 51°56′24.9″N, 14°30′39.9″E) placed on a 2% malt agar dish. Fungal biomass was obtained from a fungal pure culture grown in 2.5% malt extract medium under submerged conditions. DNA was extracted using a standard cetyltrimethylammonium bromide (CTAB)-based protocol. The fungal genome was sequenced using an Ion Torrent PGM (Ion PGM sequencing 200 kit version 2, 318 v2 Chip) and generated a 200-bp fragment library (Ion Xpress Plus fragment library kit; Thermo Fisher, Darmstadt, Germany). Before assembly, reads were filtered to include only lengths of 140 to 260 bp. Five million reads were then assembled using MIRA4.0 (8), and the Geneious R10 assembler (9) was used to filter duplicate contigs. The assembly included 826 contigs (maximum length, 505,685 bp) with a total length of 47.7 Mb. Altogether, 12,795 protein-coding genes were predicted using AUGUSTUS version 3.2.2 (fungal data set, Ascomycota_*odb9*; species parameter, Botrytis cinerea) (10). Specific enzymes of interest were annotated and filtered using Blast2GO (BioBam, Valencia, Spain) or identified in the genome using Blastp (matrix, BLOSUM62; E value, 1e^−1^) searches with known crystal structure-based reference sequences. Genome single-copy ortholog analysis performed with BUSCO version 2.0 (11) reported a completeness of 97.6%. Quality statistics using QUAST version 4.5 (12) calculated an *N*_50_ value of 114,694 bp and an average G+C content of 41.7%. Prediction of carbohydrate-active enzymes (CAZymes) (dbCAN [13]) resulted in 679 entries (Table 1). Genes encoding ligninolytic class II peroxidases (e.g., manganese or lignin peroxidases) typically secreted by white-rot fungi were not found. On the other hand, several other heme-containing peroxidases were identified (14) (Table 1). Furthermore, genes encoding peroxiredoxins (thioredoxin-dependent thiol peroxidases), which are probably involved in the removal of reactive oxygen species and thereby facilitate phytopathogenic or saprotrophic processes, could be detected (Table 1).

**TABLE 1:** CAZyme classes and enzymes of interest detected in the genome of DSM 105466

Enzyme or domain group^*a*^	No. of proteins	GenPept accession no.
Enzyme class		
Glycoside hydrolases	286	
Glycosyl transferases	98	
Polysaccharide lyases	6	
Carbohydrate esterases	125	
AA enzymes	99	
Lytic polysaccharide monooxygenases (AA9)	5	RFU24664, RFU34529, RFU33477, RFU29943, RFU26461
Associated modules		
CBM	65	
Cellulose-binding domain CBM1	19	
Enzymes of interest		
Unspecific peroxygenases	2	RFU33006, RFU31720
Dye-decolorizing peroxidases	1	RFU31715
Thioredoxin-dependent thiol peroxidases	4	RFU29069, RFU28123, RFU27935, RFU27120

a AA, auxiliary activity; CBM, carbohydrate-binding module.

### Data availability.

This whole-genome shotgun project has been deposited at DDBJ/ENA/GenBank under the accession number NCSJ00000000. The version described in this paper is version NCSJ02000000. The Sequence Read Archive (SRA) number is SRR5434225.
